# How does the brain do more with less energy?

**DOI:** 10.1093/nsr/nwag634

**Published:** 2026-10-14

**Authors:** Yongjun Wang

**Affiliations:** China National Clinical Research Center for Neurological Diseases, China; Department of Neurology, Beijing Tiantan Hospital, Capital Medical University, China

The brain is undoubtedly one of the most remarkable intelligent systems in nature. The human brain supports perception, memory, learning, thinking and motion with a mere 20 W of metabolic power [1]. In contrast, artificial intelligence often requires enormous computing resources and energy. Although neuronal communication, brain oscillations, neural connectivity and large-scale activity have been extensively described [2–4], it remains unclear why the brain works so efficiently.

In a recent pioneering study published in *National Science Review* [5], Jinxuan Ma and Wanlin Guo at Nanjing University of Aeronautics and Astronautics proposed an energy-minimized neural sphere (NS) to model the information-handling dynamics of the brain. They abstract functionally related local circuits into neural ensembles (NEs), convert biological neurons into digital neurons that retain morphological and electrophysiological information and optimize neuronal distribution under constraints of non-overlapping, metabolic space, topology connection and morphology of neuron. The energy-optimal arrangement tends to be spherical, defining the NS (Fig. 1a). By interconnecting many small-scale NSs, the authors simulate the electrophysiological activities of NEs with more complex functions or even the whole brain.

**Figure 1.**
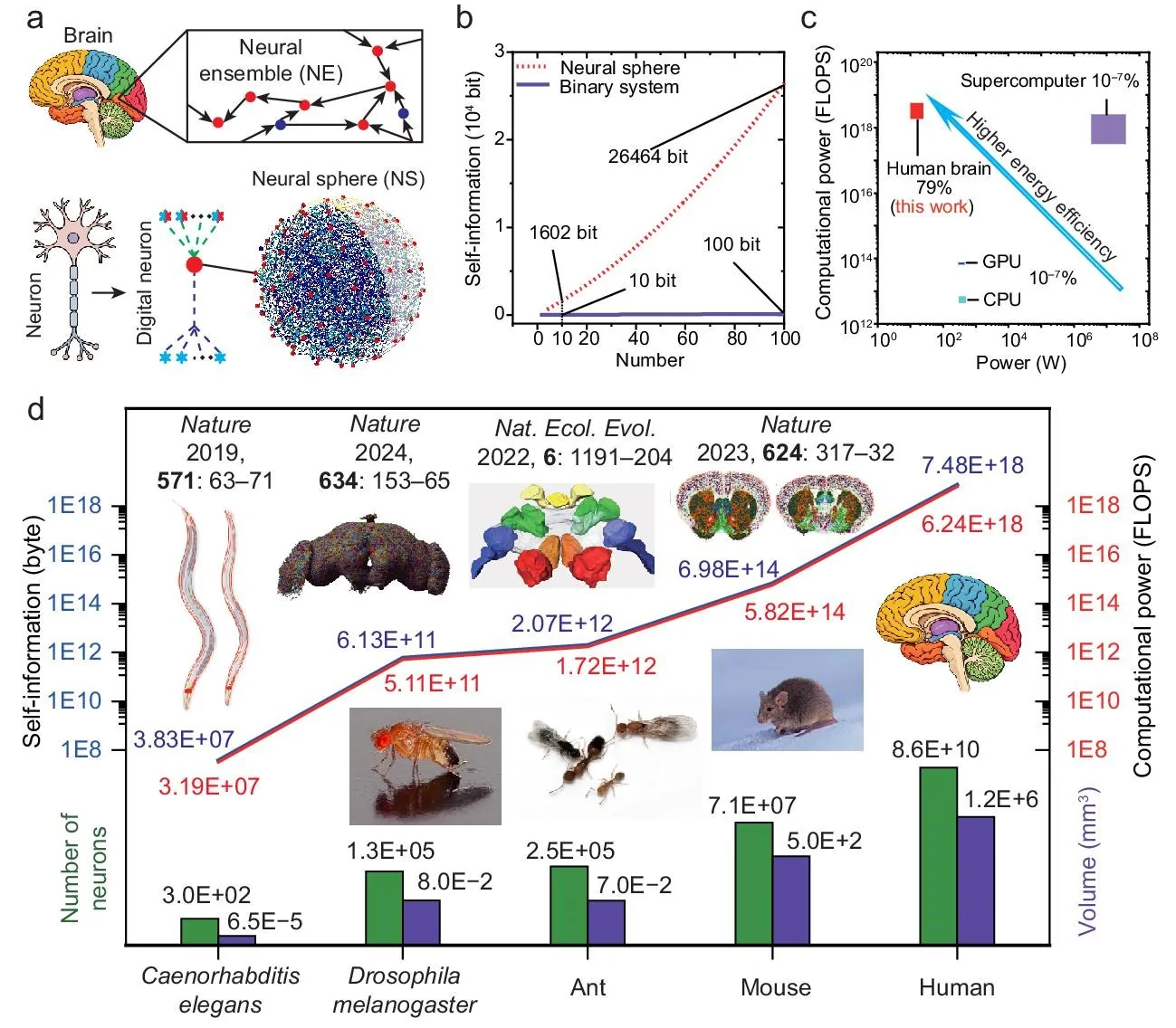
(a) Schematic of the neural sphere (NS). (b) Curves of the self-information of NS and binary system as a function of the number of neurons or devices. (c) Comparison of computational efficiency, power and energy efficiency between the human brain and silicon-based devices. The computing power is measured by double-precision floating-point operations per second. The respective energy efficiency is annotated in the figure. (d) Evaluation of the maximum storage capacity and computational power for five species by NS. Adapted from ref. [5] with permission.

Systematic simulations show that NSs exhibit four typical activities: briefly triggering a few action potentials, sustained short-period, sustained long-period, and random potential activity. For example, a 10-neuron fully positive feedback NS displays an ultra-long period of 288.11 ms and an average frequency of 167 Hz, within the fast gamma band. Most NSs with connection probabilities between 40% and 70% exhibit ultra-long period activity, suggesting a regime that can store ordered information. Using chaos dynamics and fractal theory, the authors argue that the brain memorizes and processes information through electrophysiological strange attractors formed by the global cooperation of neurons in the spatiotemporal structure, different from linear superposition based on synaptic polymorphism or the all-or-nothing of action potentials.

Under this framework, the brain substantially outperforms binary systems in self-information and energy efficiency (Fig. 1b and c). Estimates across organisms suggest that the human brain has reached an unparalleled level of natural intelligence (Fig. 1d). Previous theories assumed that the brain memorizes information linearly in synapses or neurons, and the maximum storage estimate was 1 × 10^15^ Bytes [6]. In contrast, the results for the human brain obtained through this new nonlinear framework are truly astonishing: the maximum storage capacity is predicted to be 7.48 × 10^18^ Byte—about three orders of magnitude higher than previous estimates, and the maximum computational power is 6.24 × 10^18^ FLOPS, equivalent to ∼78 000 modern GPUs. Landauer analysis further suggests a physical limit of about 2.37 W, whereas the brain’s computing and communication power is about 3 W, only 1.26 times the limit, and corresponding to an energy efficiency of up to 79%. In contrast, current advanced chips exceed their Landauer limit by about 10^9^ times, with efficiencies near 10^−7^%. This means that, after long-term evolution, the human brain may have almost reached its energy-efficiency limit. There is therefore significant potential for improving the energy efficiency of current semiconductor chip technology.

Over the past decades, large-scale initiatives such as the European Human Brain Project have generated vast amounts of data and greatly advanced our understanding of the brain [7]. Nevertheless, existing studies remain constrained by conceptual and methodological limitations and have yet to reveal the physical mechanisms underlying natural intelligence. In summary, Ma and Guo [5] provide an inspiring physical picture for understanding how natural intelligence processes information so efficiently, and this theory may open new possibilities for developing more efficient brain-inspired intelligence architectures.

## References

[1] Howarth C, Gleeson P, Attwell D. J Cereb Blood Flow Metab 2012; 32: 1222–32. 10.1038/jcbfm.2012.35 PMC3390818 22434069

[2] Laughlin SB, Sejnowski TJ. Science 2003; 301: 1870–4. 10.1126/science.1089662 PMC2930149 14512617

[3] Turner NL, Macrina T, Bae JA et al. Cell 2022; 185: 1082–100. 10.1016/j.cell.2022.01.023 PMC9337909 35216674

[4] Luo L . Science 2021; 373: eabg7285. 10.1126/science.abg7285 PMC8916593 34516844

[5] Ma J, Guo W. Natl Sci Rev 2026; 13: nwag373. 10.1093/nsr/nwag373 PMC13455624 42577833

[6] Turing AM . Mind 1950; LIX: 433–60. 10.1093/mind/LIX.236.433

[7] Naddaf M . Nature 2023; 620: 718–20. 10.1038/d41586-023-02600-x

